# The level of genetic diversity and differentiation of tropical lotus, *Nelumbo nucifera* Gaertn. (Nelumbonaceae) from Australia, India, and Thailand

**DOI:** 10.1186/s40529-020-00293-3

**Published:** 2020-05-16

**Authors:** Yeshitila Mekbib, Shi-Xu Huang, Boniface K. Ngarega, Zhi-Zhong Li, Tao Shi, Ke-Fang Ou, Yu-Ting Liang, Jin-Ming Chen, Xing-Yu Yang

**Affiliations:** 1 CAS Key Laboratory of Aquatic Botany and Watershed Ecology, Wuhan Botanical Garden, Chinese Academy of Sciences, Wuhan, 430074 China; 2 Center of Conservation Biology, Core Botanical Gardens, Chinese Academy of Sciences, Wuhan, 430074 China; 3 University of Chinese Academy of Sciences, Beijing, 100049 China; 4 Ethiopian Biodiversity Institute, P.O.Box 30726, Addis Ababa, Ethiopia; 5 College of Life Science, South-Central University for Nationalities, Wuhan, 430074 China; 6 Wuhan Institute of Landscape Architecture, Wuhan, 430081 China

**Keywords:** Conservation, Genetic diversity, Gene flow, *Nelumbo nucifera*, SSR markers, Tropical lotus

## Abstract

**Background:**

*Nelumbo nucifera* Gaertn., a perennial aquatic macrophyte species, has been cultivated in several Asian countries for its economic importance, and medicinal uses. Two distinct ecotypes of the species are recognized based on the geographical location where the genotypes are adapted, i.e., tropical lotus and temperate lotus. The genetic diversity levels and differentiation of the tropical lotus from poorly studied geographic regions still remain unclear. Here, the population genetic diversity and structure of 15 tropical lotus populations sampled from the previous understudied natural distribution ranges, including India, Thailand, and Australia, were assessed using nine polymorphic SSR markers.

**Results:**

The SSR markers used to genotype the 216 individuals yielded 65 alleles. The highest and lowest genetic diversity estimates were found in Thailand and Indian populations, respectively. STRUCTURE analysis revealed three distinct genetic clusters, with relatively low admixtures, supported by PCoA cluster analysis. Low levels of gene flow (mean N⁠m = 0.346) among the three genetic clusters signified the Mantel test for isolation by distance, revealing the existence of a positive correlation between the genetic and geographic distances (r = 0.448, *P *= 0.004). Besides, AMOVA analysis revealed a higher variation among populations (59.98%) of the three groups. Overall, the populations used in this study exposed a high level of genetic differentiation (F_ST_ = 0.596).

**Conclusions:**

The nine polymorphic microsatellite markers used in our study sufficiently differentiated the fifteen tropical *N. nucifera* populations based on geography. These populations presented different genetic variability, thereby confirming that populations found in each country are unique. The low genetic diversity (H_E_ = 0.245) could be explained by limited gene flow and clonal propagation. Conserving the available diversity using various conservation approaches is essential to enable the continued utilization of this economically important crop species. We, therefore, propose that complementary conservation approaches ought to be introduced to conserve tropical lotus, depending on the genetic variations and threat levels in populations.

## Background

*Nelumbo nucifera* Gaertn. (Lotus), a perennial aquatic macrophyte species, belongs to the genus *Nelumbo* in the family Nelumbonaceae. Cultivation of lotus dates long back in history as an ornamental and vegetable in several Asian countries (Guo 2009; Yang et al. 2012; Zhang et al. 2014). *N. nucifera* is mainly distributed in Asia and Australia (Han et al. 2007), and has also been utilized for its economical importance (Yang et al. 2013). In China, for example, *N. nucifera* seeds are widely used for the preparation of Chinese herbal medicine (Chen et al. 2008; Li et al. 2010), and the rhizome of this species is a common vegetable (Tian et al. 2008). *N*. *nucifera* flowers are the main traditional flowers in China, while in India and Vietnam, they are regarded as the national flowers (Chen et al. 2008; Tian et al. 2014).

Lotus flowers are protogynous and usually out-crossed by insects (Kubo et al. 2009). This species can be propagated either by seeds or rhizomes (Goel et al. 2001; Pan et al. 2011). Lotus is capable of producing new hybrids through hybridization between wild and domesticated varieties (Liu et al. 2012). So far, a sizable number of cultivars have been developed from *N. nucifera* (Li et al. 2015). Notably, the wild lotus populations have served as essential germplasm sources for breeding purposes (Xue et al. 2006; Han et al. 2007), and varied agro-climatic conditions have contributed to the existence of diverse genotypes of wild lotus in China (Liu et al. 2012).

Recently, morphological features, ecological adaptation, and genetic studies in lotus indicated that the South-eastern Asia lotus is distinct from Chinese lotus (Li et al. 2010). Zhang and Wang (2006) grouped the *N. nucifera* populations into two distinct ecotypes based on the geographical location where the genotypes are adapted, i.e., tropical lotus and temperate lotus. These ecotypes have shown differences in the duration of flowering, growth, and rhizome morphology. The temperate lotus have annual growth habits and big rhizome, whereas the tropical lotus is perennial, has a small rhizome and long flowering period (Zhang and Wang 2006). Lotus grown in East and North-east Asian countries belong to the temperate group, whereas the lotus grown in South-east Asian countries and Australia are considered as tropical ecotype (Zhang and Wang 2006; Li et al. 2010). A previous study revealed that the Thailand lotus, one of the tropical lotus groups, had 2 to 3 months longer flowering periods than the Chinese cultivars (Li et al. 2010; Yang et al. 2013). Tropical lotus is often used for enhancing the ornamental value of temperate lotus by providing valuable traits for developing varieties with a more extended flowering period (Li et al. 2010; Liu et al. 2012; Yang et al. 2013).

Future breeding programs and conservation of *N. nucifera* will depend on the available knowledge of genetic variation among populations (Han et al. 2009; Hu et al. 2012). In addition, genetic diversity and structure studies avail platforms for undertaking evidence-based management planning (Luo et al. 2018). Previous studies have assessed the genetic diversity of *N. nucifera* (Han et al. 2009; Pan et al. 2011), with much consideration being accorded to the temperate lotus. These studies have revealed higher genetic diversity levels for *N. nucifera* using varied molecular markers (Na et al. 2009; Han et al. 2009; Pan et al. 2011). On the contrary, the population genetic studies on tropical lotus have mostly utilized lotus populations from Thailand, however, with relatively low sampling (Li et al. 2010; Hu et al. 2012). Comparing the genetic diversity levels of the two ecotypes yields striking results. For instance, Liu et al. (2012) indicated that tropical lotus had lower genetic diversity than temperate lotus. However, a more recent study by Yang et al. (2013) showed that the wild tropical lotus had higher genetic diversity than the temperate ecotype. Hu et al. (2012) also reported that the natural lotus accessions from Thailand differentiated from other natural lotus accessions in South-east Asian countries and China using variable molecular markers (AFLPs and SSRs). Among these studies, only a few samples of the tropical lotus were included, and the representations of the tropical lotus were insignificant in comparison to temperate groups. To this day, the genetic diversity of the tropical *N. nucifera* ecotypes has not explicitly been addressed from the other major distribution regions, including India and Australia, compared to Thailand populations. The genetic diversity levels and differentiation of the tropical lotus from these poorly studied geographic regions remain unclear. Therefore, there is the need to conduct population genetic studies of tropical lotus from these understudied areas.

Here, we genotyped 15 tropical *N. nucifera* populations sampled from the natural distribution ranges in Australia, India, and Thailand using nine polymorphic microsatellite markers. We aim to (i) evaluate the level of genetic diversity of the tropical lotus populations from the previous poorly studied natural distribution ranges, and (ii) estimate the degree of differentiation and population structure of *N. nucifera*.

## Methods

### Sample collections and DNA extraction

**Fig. 1:**
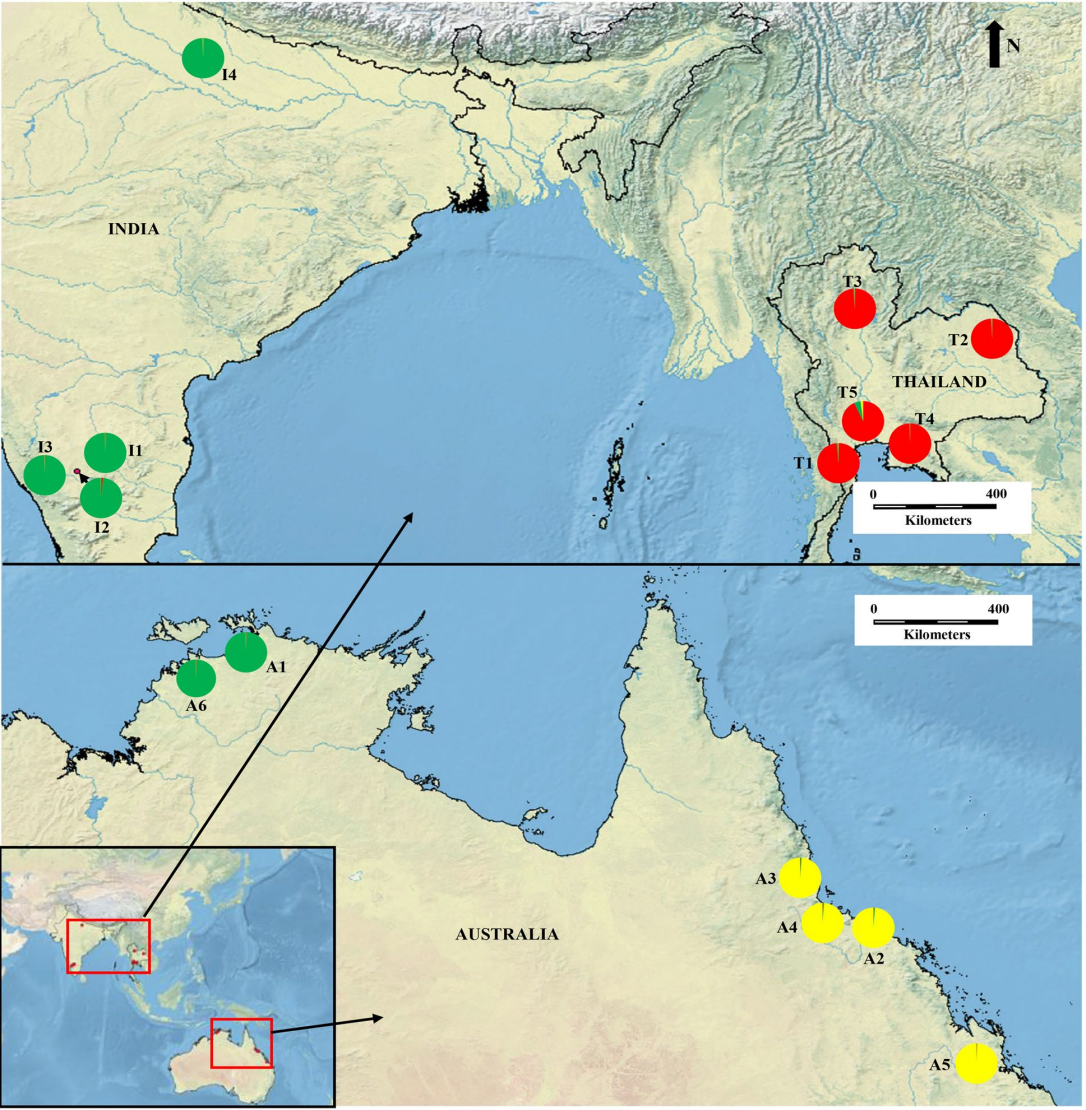
Sample collection sites of 15 *N. nucifera* tropical populations in Australia, India, and Thailand. The pie charts indicate the proportion of admixtures in the three genetic groups (K = 3), which were yielded by STRUCTURE analysis

Fifteen wild tropical *N. nucifera* populations comprising of 216 individuals were sampled from the natural distribution range in Australia, India, and Thailand (Table 1; Fig. 1). *N. nucifera* is a clonal species, and therefore, to reduce the resampling of the same individuals, leaves samples were collected at a minimum 10 m apart. The collected leaves were dried with silica gel and preserved in the refrigerator until DNA extraction. The DNA extraction and quantification followed a similar protocol as published in Islam et al. (2020), followed by preservation in a freezer at − 20 °C for subsequent analysis.

**Table 1:** Locations and sample size of *N. nucifera* populations investigated in the present study

Population	Geographic origin	Latitude (N)	Longitude (E)	Sample size
A1	Darwin, Northern territory, Australia	− 11.9500°	132.0500°	6
A2	Townswille Road, Queensland, Australia	− 19.4600°	147.3100°	23
A3	Ingham wetland, Queensland, Australia	− 18.6550°	146.1530°	18
A4	Ross River, Townswille, Queensland, Australia	− 19.3257°	146.7278°	8
A5	Rockhampton, Queensland, Australia	− 23.2570°	150.3930°	15
A6	Darwin, Northern territory, Australia	− 12.5630°	131.3040°	3
I1	Bangalore-Mysore road, Ramanagara, Karnataka, India	12.7810°	77.3690°	15
I2	Bangalore-Mysore road, Mandy, Karnataka, India	12.5275°	76.8910°	16
I3	Heggadadevanakote to Saraguru main road, Mysore, Karnataka, India	12.0742°	76.3445°	16
I4	Hardoi, Uttar Pradesh, India	27.1617°	80.3275°	16
T1	Nam rit, Mueang uttaradit, Uttaradit, Thailand	17.6857°	100.1410°	16
T2	Map pong, Phan thong, Chon buri, Thailand	13.4323°	101.1305°	16
T3	Nong pla lai, Khao yoi, Phetchaburi, Thailand	13.1817°	99.8677°	15
T4	Somdet/Lam huai lua, Somdet, Kalasin, Thailand	16.6807°	103.7350°	17
T5	Nongo, Ban pong, Ratchaburi, Thailand	13.7930°	99.9510°	16

### SSR genotyping and PCR amplifications

Nine SSR markers previously developed for *N. nucifera* by Tian et al. (2008), Kubo et al. (2009), and Pan et al. (2010), were selected for the present study (Additional file 1: Table S1). Fluorescent dye FAM (Applied Biosystems, Foster City, CA, USA) was used to label all forward primers. The polymerase chain reactions (PCR) and amplifications were performed following Islam et al. (2020). PCR products were confirmed by electrophoresis on 2.0% (W/V) agarose gel stained with ethidium bromide. Later, ABI 3730 XL automated sequencer (Wuhan Gene Create Biological Engineering Co. Ltd., Wuhan, China) was used to identify the products. GeneScan 500 LIZ (Applied biosystems) was used to check the dye sizes in each lane to allow the correct determination of fragment size. Lastly, the allele sizes were detected manually using GeneMarker2.2.0 (Soft Genetics) with the default settings.

### Data analysis

#### Genetic diversity indices

The Cervus version 3.0 program (Kalinowski et al. 2007) was used to assess Hardy–Weinberg Equilibrium deviations with Bonferroni corrections as well as the polymorphic information content (PIC) for each SSR marker and inbreeding coefficient (F_IS_) for the 15 *N. nucifera* populations were estimated. GenAlEx version 6.5 (Peakall and Smouse 2012) was used to determine the genetic diversity characteristics of all loci and populations. The following parameters were assessed for each SSR; the effective number of alleles (Ne), expected heterozygosity (He), observed heterozygosity (Ho), the number of alleles per locus (Na). Population-based characteristics estimated included; the effective number of alleles (N_E_), expected heterozygosity (H_E_), observed heterozygosity (H_O_), the number of alleles per locus (N_A_), Shannon’s information index (I_S_), the number of private alleles (Np), and inbreeding coefficient (F_IS_).

#### Population structure

To examine the level of genetic variation among *N. nucifera* populations, and estimates of genetic differentiation (F_ST_), the analysis of molecular variance (AMOVA) was done in Arlequin version 3.1 (Excoffier et al. 2005). STRUCTURE version 2.3.3 (Pritchard et al. 2000) that uses a Bayesian algorithm was used to assign populations to genetic clusters. 100,000 burn-in steps and ten iterations for each K from 1 to 15 were run independently (where K = Number of populations), followed by 1,000,000 Markov Chain Monte Carlo (MCMC). An online tool, STRUCTURE HARVESTER (http://taylor0.biology.ucla.edu/structureHarvester/) (Earl 2012), was used to analyze the results and predict the suitable number of genetic clusters. K consistent values were readjusted in CLUMPP version 1.1.2 (Jakobsson and Rosenberg 2007) while employing the Greedy algorithm with 10,000 replications. The resulting genetic structure of the 15 populations *N. nucifera* was constructed and displayed in DISTRUCT version 1.1 (Rosenberg 2004). Using Nei’s genetic distance matrix (Nei et al. 1983), GenAlEx version 6.5 (Peakall and Smouse 2012) executed the principal coordinate analysis (PCoA).

#### Bottleneck analysis

Bottlenecks across the 15 *N. nucifera* populations were assessed using the Bottleneck version 1.2.02 program (Piry et al. 1999). Wilcoxon’s sign-rank tests were performed with 10,000 simulations at the 5% significance, using the two-phase model (TPM = 70%SMM +30%IAM), the step-wise mutation model (SMM), and the infinite allele model (IAM). The deviation of the populations from normal L-shaped distribution (mode shift), which indicates a demographic bottleneck on populations, was also checked (Luikart and Cornuet 1998).

#### Estimation of historical gene flow

The number of migrants per generation (Nm) among the genetic groups (K = 3), in the previous 4Ne generations (Ne = effective population size), was estimated by MIGRATE-n version 3.7.2 program (Beerli 2012). A Bayesian and coalescent inference approach (Beerli 2006) was used while applying the Brownian approximation model. The θ (mutational scaled effective population size) and M (mutation scaled migration rate) were obtained from the program with settings attuned to default, then used to approximate Nm. The Nm was estimated as in the equation; ${\text{Nm }} = \left[ {\left( {\uptheta {\text{a }} \times {\text{Mb}} \to {\text{a}}} \right)/4} \right],$ i.e., population b migrants per generation to population a (Beerli 2012).

## Results

### Characteristics of the microsatellite markers

All SSRs markers observed significant Hardy–Weinberg deviations. The nine microsatellite markers used in the present study yielded high polymorphisms in all populations. For each microsatellite marker, the effective number of alleles (Ne) varied from 1.223 to 1.956 (mean = 1.475). Observed (Ho) and expected heterozygosity (He) estimates ranged from 0.067 to 0.631 and 0.140 to 0.474, respectively (mean, Ho = 0.274 and He = 0.245) (Additional file 1: Table S1). Nelumbo-13 and PR05 markers had the highest number of alleles (Na = 10). PIC is considered as a measure of the informativeness of the SSR markers (Babu et al. 2014), and high PIC values are reported to have a high discriminating ability and recommended for population genetic diversity studies (Ngailo et al. 2016). PIC values of the microsatellite markers used in our study varied from 0.322 at locus Nelumbo-32 to 0.775 at locus PR05 (mean = 0.593). Only, Nelumbo-32 and NNEST17 markers had PIC values less than 0.50.

### Genetic diversity of *N. nucifera*

Sixty-five alleles were identified in the 15 tropical *N. nucifera* populations, ranging from six to ten alleles per locus (mean = 7.220) (Additional file 1: Table S1). The number of effective alleles (N_E_) and the number of observed (N_A_) per population ranged from 1.140 to 2.023 and 1.333 to 2.667, respectively. The heterozygosity levels, observed and expected, ranged from 0.044 to 0.824 and 0.081 to 0.470, respectively. The average expected heterozygosity (H_E_ = 0.358) was higher in Thailand than in both India and Australia. Similarly, Shannon’s information index varied from 0.129 to 0.730. Private alleles were detected in nine of the 15 populations examined. Eleven of the 19 observed private alleles were found in populations sampled from Thailand, and population T4 had the highest count (6) **(**Table 2).

**Table 2:** The genetic diversity parameters measures among the 15 *N. nucifera* populations

Population	N_A_	N_E_	I_S_	H_O_	H_E_	N_P_	F_IS_
A1	1.556 (0.294)	1.383 (0.228)	0.268 (0.145)	0.074 (0.056)	0.164 (0.088)	1	0.487
A2	2.000 (0.236)	1.465 (0.155)	0.412 (0.106)	0.275 (0.115)	0.260 (0.070)	0	− 0.162
A3	2.000 (0.167)	1.470 (0.128)	0.431 (0.080)	0.296 (0.127)	0.277 (0.061)	0	− 0.132
A4	1.778 (0.278)	1.285 (0.136)	0.286 (0.107)	0.181 (0.110)	0.168 (0.066)	0	− 0.205
A5	2.111 (0.309)	1.433 (0.151)	0.400 (0.122)	0.259 (0.117)	0.238 (0.076)	3	− 0.169
A6	1.444 (0.242)	1.311 (0.157)	0.244 (0.124)	0.185 (0.126)	0.160 (0.080)	1	0.111
I1	1.778 (0.278)	1.358 (0.180)	0.302 (0.124)	0.044 (0.029)	0.183 (0.078)	1	0.693
I2	1.778 (0.278)	1.402 (0.161)	0.349 (0.124)	0.063 (0.090)	0.216 (0.079)	0	0.668
I3	1.333 (0.167)	1.140 (0.108)	0.129 (0.126)	0.090 (0.090)	0.081 (0.055)	0	− 0.291
I4	1.556 (0.294)	1.251 (0.126)	0.229 (0.078)	0.194 (0.097)	0.143 (0.072)	2	− 0.371
T1	2.667 (0.236)	1.661 (0.160)	0.598 (0.116)	0.465 (0.127)	0.353 (0.060)	2	− 0.438
T2	2.222 (0.364)	1.494 (0.190)	0.437 (0.091)	0.264 (0.130)	0.254 (0.078)	1	− 0.172
T3	2.333 (0.408)	1.780 (0.228)	0.574 (0.127)	0.415 (0.161)	0.358 (0.083)	0	− 0.236
T4	2.667 (0.333)	2.023 (0.150)	0.730 (0.138)	0.824 (0.112)	0.470 (0.062)	6	− 0.771
T5	2.111 (0.222)	1.673 (0.161)	0.539 (0.102)	0.479 (0.154)	0.357 (0.068)	2	− 0.367
Mean	1.956 (0.077)	1.475 (0.045)	0.395 (0.103)	0.274 (0.033)	0.245 (0.020)	1.270	

*N*_*A*_ observed number of alleles, *N*_*E*_ effective number of alleles, *I*_*S*_ Shannon’s information index, *H*_*O*_ observed heterozygosity, *H*_*E*_ expected heterozygosity, *F*_*IS*_ coefficient of inbreeding, *N*_*P*_ number of private alleles

Eleven populations showed low levels of coefficient of inbreeding (F_IS_). This observation reflects the presence of high cross-pollination levels among populations. However, only four populations (A1, A6, I1, and I6) had positive F_IS_ values, suggesting that there existed inbreeding among the individuals of these populations. Populations T4 and I3 had the highest and lowest genetic diversity (H_E_), respectively. Overall, the microsatellite markers showed a low genetic variation in *N. nucifera* populations.

### Genetic structure of *N. nucifera*

**Fig. 2:**
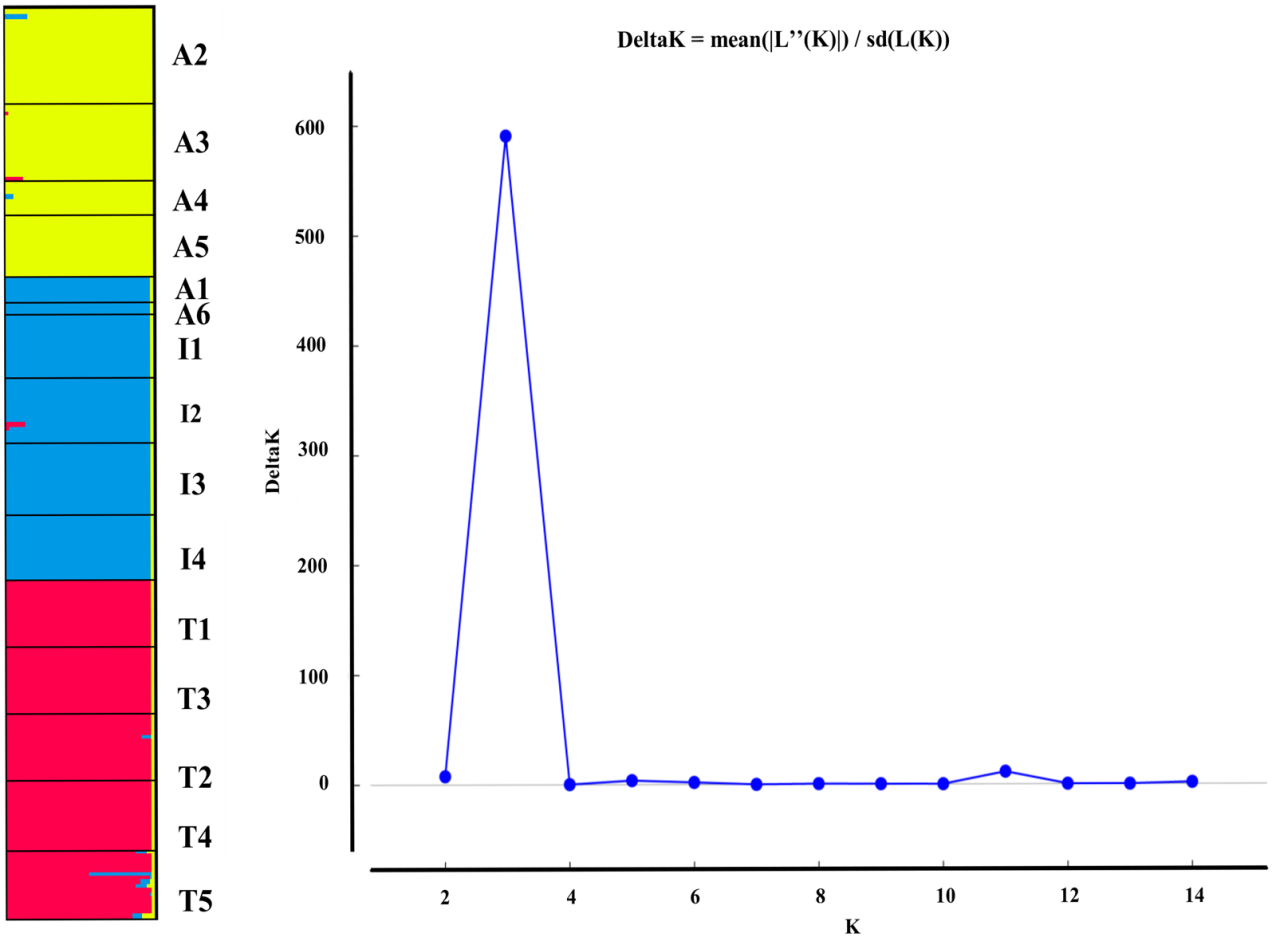
Genetic structuring of the15 tropical *N. nucifera* populations obtained from the STRUCTURE analysis, K = 3 (shown on the left); and the plot of K against delta K (shown on the right)

**Fig. 3:**
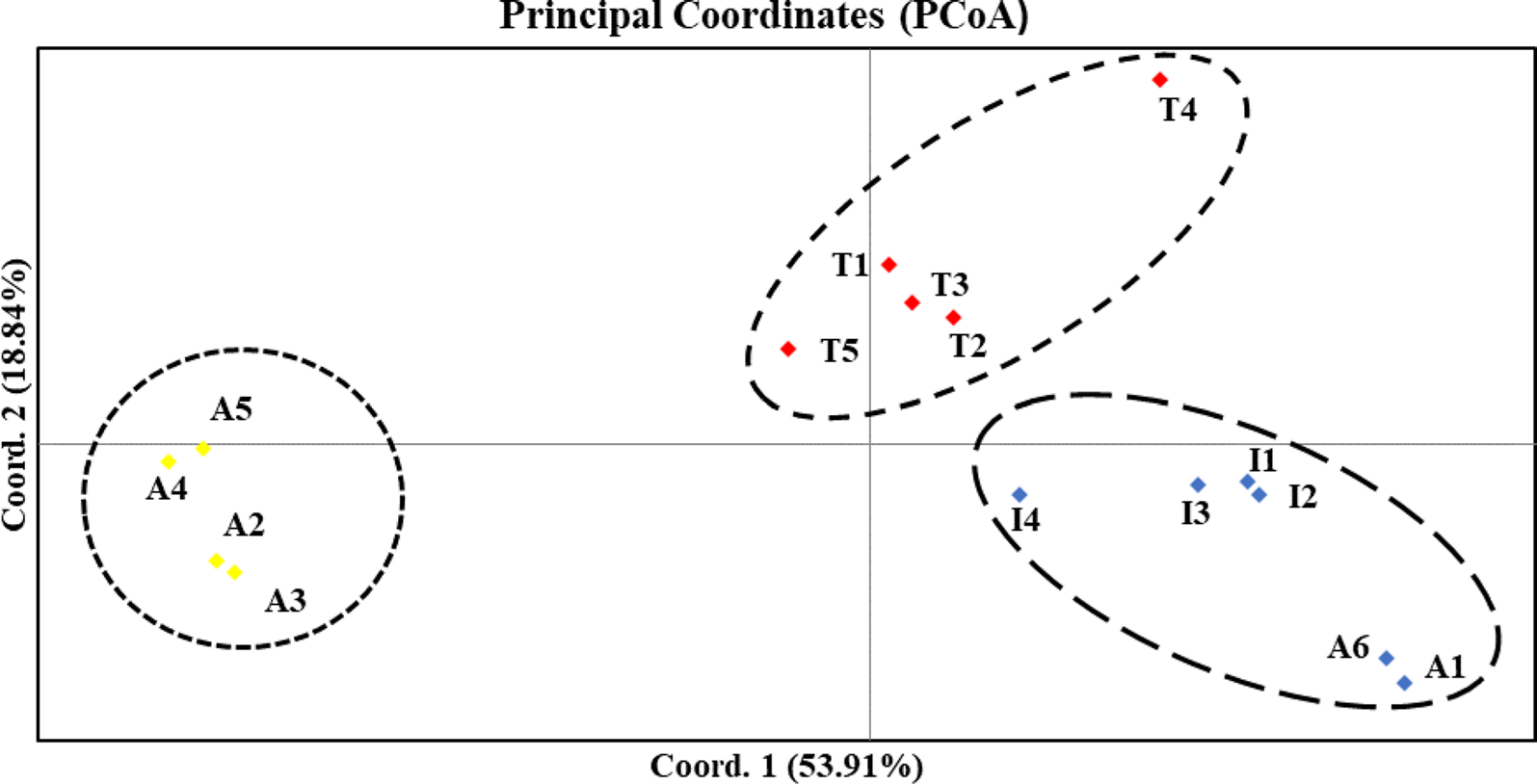
The scatter plot of principal coordinate analysis (PCoA) based on the microsatellite data. Australia populations (A), India populations (I), and Thailand populations (T). Coord.1 (53.91%) and Coord. 2 (18.84%) refer to the first and second principal components, respectively

The Bayesian clustering in STRUCTURE suggested three genetic clusters in the *N. nucifera* populations, according to delta *K*. These populations were divided geographically according to the three countries (India, Thailand, and Australia), except for two Australian populations that were assigned together with the Indian populations (Fig. 2). Among the 15 tropical *N. nucifera* populations, the highest (2.383) and lowest (0.005) genetic distance was found in the populations sampled from Australia (Additional file 2: Table S2). The PCoA analysis revealed similar clustering patterns as STRUCTURE results, including the assignment of the two Australian populations to the Indian cluster (Fig. 3). The first and second axes in the PCoA explained 72.75% of the total variation (Fig. 3).

**Fig. 4:**
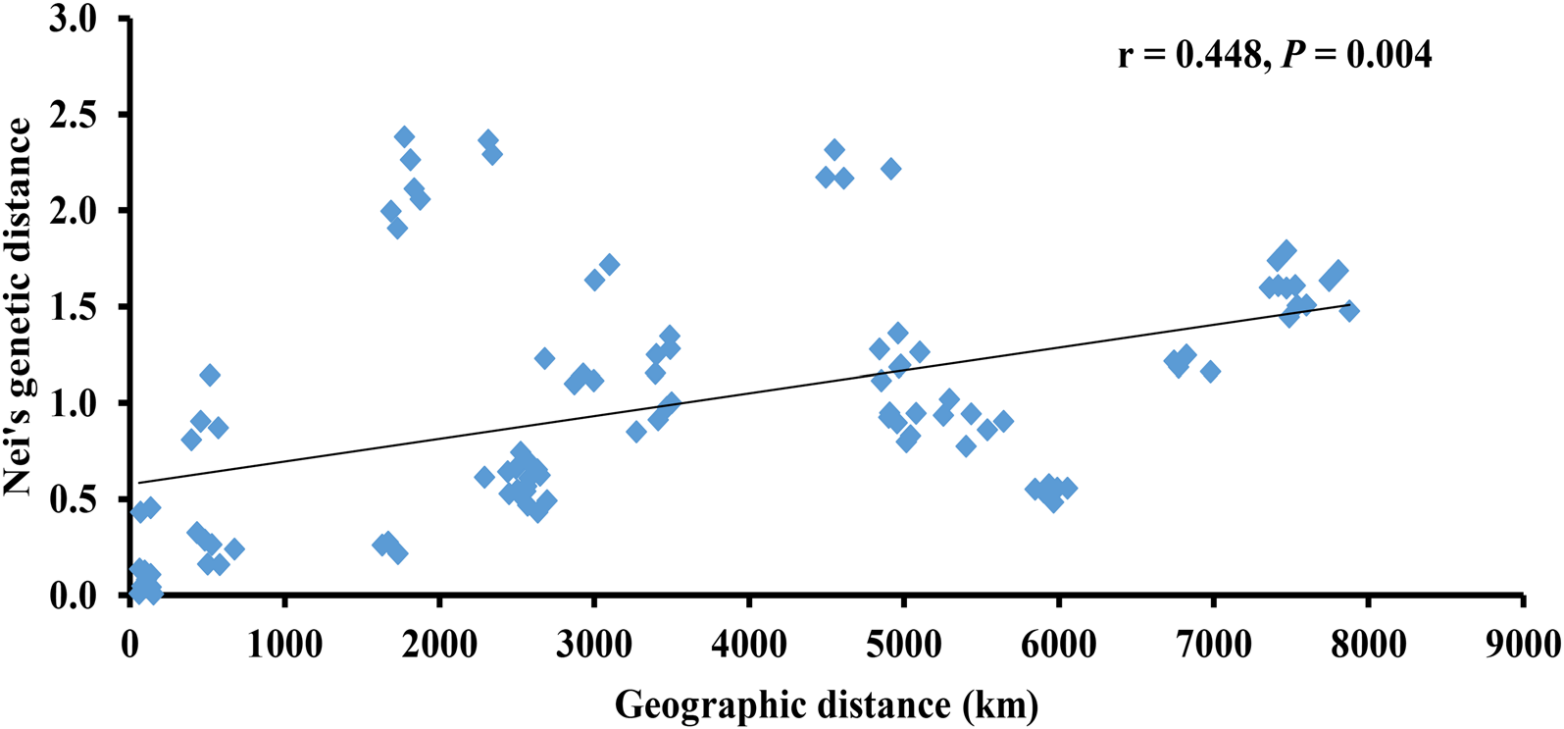
Mantel test for isolation by distance between Nei’s genetic distance and geographic distance (km) for the *N. nucifera* populations

Results of AMOVA revealed a higher variation among populations (59.98%) than within populations (40.02%) of the three countries, supported by high levels of genetic differentiation (F_ST_ = 0.596) (Table 3). In addition, the Mantel’s test confirmed the existence of a significant positive correlation between Nei’s genetic distance (Nei et al. 1983) and geographic distance (km) for all pairwise populations (r = 0.448, *P *= 0.004) (Fig. 4). Mantel test results indicated that the geographical distribution of the populations had contributed significantly to the observed genetic diversity.

**Table 3:** Analysis of molecular variance (AMOVA) for the 15 *N. nucifera* populations

Source of variation	d.f.	Sum of squares	Variance components	PV (%)	F_ST_	P
1. Total variations						
Among all populations	14	621.849	1.519	59.98	0.596	< 0.001
Within population	417	422.755	1.013	40.02		< 0.001
2. Three groups as in STRUCTURE						
Among groups	2	370.894	1.125	39.48	0.626	< 0.001
Among population within groups	12	250.995	0.711	24.96		< 0.001
Within population	417	422.755	1.013	35.56		< 0.001
Total	431	1044.604	2.851			

*d.f.* degree of freedom, *PV* percentage of variation

### Population demographic bottlenecks and historical gene flow

The results of the bottleneck analysis are outlined in Additional file 3: Table S3. The three models used detected recent bottlenecks in T4, whereas only the infinite allele model (IAM) detected bottleneck in T5. Similarly, five populations (A1, A6, I1, I2, and I4) showed a shifted mode indicating the effect of recent population bottlenecks. The Migrate-n results of gene flow (Nm) among the three groups of *N. nucifera* populations revealed that the mutation scale effective population size (θ) for the three genetic clusters were 0.09137, 0.09711 and 0.09759, respectively. Among the populations the computed mutation scaled migration rate (M) ranged from 4.163 (M2- > 1) to 23.631 (M2- > 3). The gene flow was higher from India to Thailand populations (Nm = 0.577). Low gene flow occurred from India to Australia populations (Nm = 0.095). The study also found bidirectional gene flow among the genetic clusters. Overall, low gene flow (mean Nm = 0.394) was obtained among the three genetic clusters of *N. nucifera* populations. The details of the historical gene flow analysis among populations are shown in Additional file 4: Table S4.

## Discussion

### Genetic diversity in wild *N. nucifera* populations

In the current study, we included 15 tropical *N. nucifera* populations sampled from natural distribution ranges in Australia, India, and Thailand. The genetic diversity (H_E_) values varied from 0.160–0.277; 0.081–0.216 and 0.254–0.470, in Australia, India and Thailand populations, respectively. This highlights the presence of wide genetic variability within each genetic group. The overall findings of this study exhibited low genetic diversity levels (mean H_E_ = 0.245), which is mainly explained by low gene flow levels and clonal propagation. The result of the inbreeding coefficient analysis revealed that A1, A2, I1, and I2 populations had significant positive F_IS_ values, which may be attributed to the high level of matings between closely related individuals. This phenomenon might have contributed to the observed low level of genetic diversity in these populations. The mean expected heterozygosity found in this study is greater than that recorded for the tropical lotus (H_E_ = 0.152) by Liu et al. (2012). This value is much lower than the value (H_E_ = 0.320) reported for the same ecotype by Yang et al. (2013). Moreover, the genetic diversity level found in this study is lower than the values reported for other aquatic plant species such as *Ottelia acuminata* (H_E_ = 0.351) by Zhai et al. (2018) and *Ottelia acuminata* var. *jingxiensis* (H_E_ = 0.441) by Li et al. (2019) using microsatellites markers. Comparably, the highest genetic diversity estimate (H_E_) in this study was found among the populations from Thailand (H_E_ = 0.360), whereas the least genetic diversity was found in Indian populations (H_E_ = 0.156). The higher genetic diversity found in Thailand populations might be related to the inherent broad genetic base of the germplasm or the presence of suitable growing conditions for the species in this country. The highest genetic diversity values (H_E_ = 0.470), was found in T4. From the result, we can suggest that the higher genetic diversity level revealed in this population might have been accumulated during a long evolutionary history of the population. The highest genetic diversity and private alleles (H_E_ = 0.470 and N_P_ = 6, respectively), were found in T4 (Table 2). From the result, we can suggest that the higher genetic diversity level revealed in this population might have been accumulated during a long evolutionary history of the population. Besides, a previous study reported that, if a population had a large size, stable, and persisted for a long period, that population could still maintain high genetic diversity even after experiencing bottleneck events (Assis et al. 2013), as evidenced in T4.

The mean PIC value (0.593) detected in this study indicates that the markers are effective for population genetic studies of *N. nucifera*. The value is comparable with the PIC value reported by Liu et al. (2012), which observed a mean value of 0.537. Recently, the study conducted by Islam et al. (2020) on *N. lutea* sampled from the USA revealed higher PIC values (0.793), generally greater than the current study PIC. Overall, the present results portrayed that the SSR markers used are handy for genetic diversity studies in the *N. nucifera* germplasm.

Unlike sexual reproduction, in clonal propagation, there is no genetic recombination, and only a rhizome is used as a seed (Chen et al. 2008). Therefore, a cultivar’s total heterozygosity remains the same when using the same rhizomes vegetative propagation. As a result, plants propagated by clonal methods generally have low genetic variation than sexually propagated ones (Chen et al. 2008; Xue et al. 2006). Li et al. (2015) inferred that asexual reproduction through rhizomes in *N. nucifera* contributed to low genetic diversity. Positive F_IS_ values observed in four *N. nucifera* populations (Table 2) reflect the presence of excess homozygote individuals, and it is expected to contribute to the lower genetic diversity in these populations, an aspect supported by Hyten et al. (2006) study. Similarly, Beatty and Provan (2011) stated that the habitat of species found at the peripheral areas are highly fragmented, and the populations are often found at the edge of their ranges. In the present study, most of the *N. nucifera* populations were sampled from the peripheral areas, for instance, the Australian populations and some Indian populations. Therefore, it is likely that these populations had already been affected by habitat fragmentation, which eventually leads to lower genetic diversity.

### Population genetic structure

The higher percentage of variation in *N. nucifera* was found among, compared to within populations of the three countries; however, this dissimilarity was not significant. The PCoA investigation revealed that *N. nucifera* populations were distinct. Similarly, the STRUCTURE analysis showed three distinct genetic clusters with low admixtures, supported by PCoA cluster analysis (Fig. 2). A1 and A6 consistently clustered together with the Indian populations. This clustering pattern is difficult to explain in terms of the proximity of geographical distance. However, we infer that the populations either might have diverged a long time ago from the same ancestors or recently introduced by humans. Xue et al. 2006 suggested that birds can occasionally disperse seeds. The geographic distance between A1 and A6 populations is approximately 106 km, and gene flow can occur between these populations. Because of this, the populations might have possessed common ancestral polymorphism, which differentiates them from other Australian populations. The low sampling of the two populations might have also contributed to the observed clustering pattern. The high level of differentiation (F_ST_ = 0.596) in the present study is lower than the previous findings reported for *N. nucifera* (Han et al. 2007; Pan et al. 2011). A recent study by Islam et al. (2020) identified a lower level of gene flow, founder effect, inbreeding, and common ancestry as the major reasons for genetic differentiation in *N. lutea* populations in the USA. Slatkin (1987) reported that a lower gene flow (less than one) can cause genetic differentiation among populations. Hence, the high F_ST_ (0.596) and the low gene flow (0.346) found in this study contributed to the observed genetic structure, supported by significant IBD patterns in the study area (r = 0.448, *P *= 0.004). Zhang et al. (2019) submitted that asexual propagation would also reduce genetic differences among individuals within populations and increase differences among populations.

### Gene flow estimation and bottleneck analysis

Gene flow may have a significant impact on the genetic differentiation of the local populations (Storfer 1999). It plays a vital role in influencing genetic variations within populations by limiting inbreeding depression (Robledo-Arnuncio et al. 2014). Results of Migrate-n analysis indicated that the highest gene flow (Nm = 0.577) was observed from India to Thailand, and the lowest (Nm = 0.095) was from India to Australia. The agents of gene flow in lotus can be insects, birds, water currents (Kubo et al. 2009; Xue et al. 2006), and humans. Besides, due to the large geographical distance between Thailand and India, gene flow may not be carried out by insects attributed to the insect’s short flight ranges. Therefore, water currents, birds or anthropogenic introductions may be significant among the main drivers of gene flow in lotus between the two countries. Slatkin (1987) indicated that genetic drift results in higher genetic differentiation when the gene flow among populations is less than one (Nm < 1). We, therefore, suggest that genetic drift might have influenced the observed genetic differentiation in the *N. nucifera* populations hence the low gene flow. Li et al. (2010) also reported a low level of recurrent gene flow among the wild populations of *N. nucifera* sampled from China, Japan, India, and Thailand. The bottleneck analysis revealed that seven of the 15 *N. nucifera* populations had experienced bottlenecks, of which, T4 and T5 had significant probabilities (Additional file 3: Table S3). Chen et al. (2019) outlined that habitat loss, fragmentation, and over-exploitation were the major factors that contributed to bottlenecks in *N. nucifera* populations. Hence, from the observation made in the present study, we presume that some of the populations (T4 and T5) have already been affected by fragmentation.

### Implication for conservations

The presence of high genetic diversity (H_E_) within crop species plays a critical role in crop improvement programs (Salgotra et al. 2015). Besides, genetic diversity determines the potential of species survival and adaptation in the changing environmental conditions (Otálora et al. 2015; Chen et al. 2019). The lotus varieties currently found under production were obtained by continuous selection from the wide diversity available in the agricultural fields and wild states (Tian et al. 2008). According to Hu et al. (2012), wild *N. nucifera* populations found in Thailand and northeastern China are valuable germplasm in lotus breeding work. In another study, it was reported that the tropical lotus germplasm found in Thailand was used in breeding to improve the ornamental and economic values of Chinese lotus varieties (Yang et al. 2013). Notably, breeders used the genetic variation found in wild species to identify agriculturally important traits and introducing them into new varieties (Samiei et al. 2010). This suggests that countries are in one way or the other dependent on other countries’ genetic resources for improving their indigenous species. At present, our study realized low genetic variability in most populations. The wetlands used as habitat for tropical lotus have been turned into agriculture, and other land uses (La-ongsri et al. 2009). This phenomenon will likely affect the populations, and the genetic diversity might continue to decline. Two (T4 and T5) out of the seven populations that had experienced recent bottlenecks had significant probabilities, indicating that anthropogenic and natural factors had already threatened them. Hence, conservation of these threatened tropical lotus germplasm deserves special attention to ensure their continued availability. The highly diverse populations found in this study could be valuable germplasm for future breeding programs of the crop. Conservation priority should be given to populations with the highest genetic diversity, and those that have exhibited recent bottlenecks. Hence, we suggest the implementation of complementary conservation (i.e., in situ and ex situ) approaches for this species.

## Conclusions

Population genetic structure studies of *N. nucifera* are essential to identify populations with unique traits and design appropriate conservation methods. The nine polymorphic microsatellite markers used in our study sufficiently differentiated the 15 tropical *N. nucifera* populations based on geography. The populations showed different genetic variability, and the results confirmed that the populations found in each country are unique. Geographically separated populations will likely develop genetic differences due to the adaptation to different habitats. We recommend that future breeding programs and conservation of *N. nucifera*, to utilize the germplasms of tropical populations with high genetic levels, as yielded in our study.

Further studies using additional samples from all the species distribution areas and more markers should be conducted to gain more insights into the population genetic structure of *N. nucifera.* Conserving the available diversity using various conservation approaches is essential to enable the continued utilization of this economically important crop species. Therefore, based on the findings of this study, conservation priority should be given to populations with a high level of genetic diversity (e.g., T4, in Thailand), and to those that have exhibited bottlenecks. We recommend that complementary conservation approaches should be effected to maintain endangered and the declining populations of tropical lotus.

## Supplementary information

**Additional file 1: Table S1.** Polymorphism information of the nine SSRs markers used in the present study.

**Additional file 2: Table S2.** Pairwise fixation index (F_ST_) values between populations of tropical *N. nucifera* (below diagonal), and Nei genetic distance (above diagonal).

**Additional file 3: Table S3.** Bottleneck analysis in 15 tropical *N. nucifera* populations.

**Additional file 4: Table S4.** Migrate-n results of historical gene flow among the three genetic groups.

## Data Availability

All data generated or analyzed during this study are included in this published article and its additional files.

## Abbreviations

AMOVA: Analysis of molecular variance
H_E_: Expected heterozygosity
H_O_: Observed heterozygosity
F_IS_: Inbreeding coefficient
F_ST_: Genetic differentiation
HWE: Hardy–Weinberg equilibrium
IAM: The infinite allele model
IBD: Isolation by distance
I_S_: Shannon’s information index
M: Mutation scaled migration rate
MCMC: Markov chain Monte Carlo
N_A_: Number of alleles per locus
N_E_: Effective number of alleles
Nm: Historical gene flow
Np: The number of private alleles
PCoA: Principal coordinate analysis
PCR: Polymerase chain reactions
PIC: Polymorphic information content
SMM: Step-wise mutation model
SSR: Simple sequence repeats
θ: Mutational scaled effective population size
