# Supplementary material for: The level of genetic diversity and differentiation of tropical lotus, Nelumbo nucifera Gaertn. (Nelumbonaceae) from Australia, India, and Thailand
Source: Bot Stud. 2020 May 16;61:15. doi: 10.1186/s40529-020-00293-3 (PMC7229132; doi:10.1186/s40529-020-00293-3)
Supplement: Supplementary file 1 — Additional file 1: Table S1. Polymorphism information of the nine SSRs markers used in the present study. [file 40529_2020_293_MOESM1_ESM.docx]

Table S1. Polymorphism information of the nine SSRs markers used in the present study

| Locus | Na | Ne | PIC | Ho | He | HWE |
| --- | --- | --- | --- | --- | --- | --- |
| Nelumbo-13  Nelumbo-32  NSh02  NS002  NS010  PR02  PR05  PR09  NNEST17  Mean | 10  6  6  6  6  8  10  7  6  7.220 | 1.490  1.323  1.297  1.383  1.346  1.223  1.956  1.627  1.632  1.475 | 0.753  0.322  0.555  0.732  0.560  0.517  0.775  0.690  0.432  0.593 | 0.335  0.067  0.196  0.313  0.175  0.144  0.631  0.449  0.156  0.274 | 0.235  0.198  0.156  0.195  0.201  0.140  0.474  0.281  0.330  0.245 | ***  ***  ***  ***  ***  ***  ***  ***  ***  *** |

Na - Observed number of alleles; Ne - Effective number of alleles; PIC - Polymorphic information content;

Ho - Observed heterozygosity; He - Expected heterozygosity; HWE - Hardy-Weinberg Equilibrium; ***-*P<0.001*
