# Supplementary material for: The level of genetic diversity and differentiation of tropical lotus, Nelumbo nucifera Gaertn. (Nelumbonaceae) from Australia, India, and Thailand
Source: Bot Stud. 2020 May 16;61:15. doi: 10.1186/s40529-020-00293-3 (PMC7229132; doi:10.1186/s40529-020-00293-3)
Supplement: Supplementary file 2 — Additional file 2: Table S2. Pairwise fixation index (FST) values between populations of tropical N. nucifera (below diagonal), and Nei genetic distance (above diagonal). [file 40529_2020_293_MOESM2_ESM.docx]

Table S2. Pairwise fixation index (F_ST_) values between populations of tropical *N. nucifera*

(below diagonal), and Nei genetic distance (above diagonal)

|  | A1 | A2 | A3 | A4 | A5 | A6 | I1 | I2 | I3 | I4 | T1 | T2 | T3 | T4 | T5 |
| --- | --- | --- | --- | --- | --- | --- | --- | --- | --- | --- | --- | --- | --- | --- | --- |
| A1 |  | 2.114 | 1.995 | 2.383 | 2.365 | 0.082 | 0.575 | 0.556 | 0.557 | 0.905 | 1.282 | 0.978 | 1.001 | 1.719 | 1.346 |
| A2 | 0.643 |  | 0.005 | 0.136 | 0.262 | 2.058 | 1.597 | 1.608 | 1.508 | 1.248 | 1.185 | 1.361 | 1.263 | 2.167 | 0.946 |
| A3 | 0.618 | 0.010 |  | 0.124 | 0.239 | 1.906 | 1.598 | 1.609 | 1.446 | 1.218 | 1.115 | 1.279 | 1.195 | 2.173 | 0.898 |
| A4 | 0.708 | 0.124 | 0.101 |  | 0.159 | 2.264 | 1.739 | 1.790 | 1.504 | 1.186 | 0.948 | 0.926 | 0.830 | 2.315 | 0.798 |
| A5 | 0.662 | 0.198 | 0.154 | 0.159 |  | 2.291 | 1.636 | 1.688 | 1.475 | 1.163 | 0.936 | 1.019 | 0.945 | 2.216 | 0.774 |
| A6 | 0.174 | 0.638 | 0.607 | 0.698 | 0.655 |  | 0.552 | 0.535 | 0.484 | 0.860 | 1.153 | 0.851 | 0.914 | 1.638 | 1.249 |
| I1 | 0.436 | 0.564 | 0.545 | 0.632 | 0.572 | 0.409 |  | 0.010 | 0.043 | 0.261 | 0.534 | 0.608 | 0.642 | 1.100 | 0.528 |
| I2 | 0.469 | 0.547 | 0.530 | 0.620 | 0.558 | 0.452 | 0.021 |  | 0.047 | 0.275 | 0.568 | 0.652 | 0.661 | 1.150 | 0.545 |
| I3 | 0.554 | 0.611 | 0.580 | 0.651 | 0.599 | 0.564 | 0.088 | 0.108 |  | 0.216 | 0.433 | 0.492 | 0.540 | 1.114 | 0.470 |
| I4 | 0.634 | 0.528 | 0.509 | 0.550 | 0.563 | 0.606 | 0.284 | 0.291 | 0.305 |  | 0.615 | 0.623 | 0.707 | 1.231 | 0.743 |
| T1 | 0.468 | 0.395 | 0.371 | 0.386 | 0.358 | 0.443 | 0.303 | 0.310 | 0.284 | 0.328 |  | 0.286 | 0.161 | 0.808 | 0.325 |
| T2 | 0.453 | 0.498 | 0.470 | 0.433 | 0.439 | 0.449 | 0.327 | 0.338 | 0.329 | 0.381 | 0.173 |  | 0.108 | 0.906 | 0.456 |
| T3 | 0.408 | 0.431 | 0.407 | 0.366 | 0.378 | 0.390 | 0.333 | 0.326 | 0.323 | 0.391 | 0.112 | 0.100 |  | 0.872 | 0.431 |
| T4 | 0.467 | 0.434 | 0.424 | 0.485 | 0.448 | 0.464 | 0.396 | 0.389 | 0.448 | 0.436 | 0.273 | 0.324 | 0.272 |  | 1.145 |
| T5 | 0.506 | 0.366 | 0.345 | 0.378 | 0.339 | 0.483 | 0.315 | 0.283 | 0.325 | 0.382 | 0.170 | 0.251 | 0.239 | 0.321 |  |
