# Supplementary material for: The level of genetic diversity and differentiation of tropical lotus, Nelumbo nucifera Gaertn. (Nelumbonaceae) from Australia, India, and Thailand
Source: Bot Stud. 2020 May 16;61:15. doi: 10.1186/s40529-020-00293-3 (PMC7229132; doi:10.1186/s40529-020-00293-3)
Supplement: Supplementary file 3 — Additional file 3: Table S3. Bottleneck analysis in 15 tropical N. nucifera populations. [file 40529_2020_293_MOESM3_ESM.docx]

Table S3. Bottleneck analysis in 15 tropical *N. nucifera* populations

| Population | IAM | Models  SMM | TPM | Mode shift |
| --- | --- | --- | --- | --- |
| A1 | 0.250 | 0.375 | 0.375 | Shifted |
| A2 | 0.055 | 0.688 | 0.469 | Norma L-shaped |
| A3 | 0.383 | 0.742 | 0.547 | Normal L-shaped |
| A4 | 0.219 | 0.094 | 0.219 | Normal L-shaped |
| A5 | 1.000 | 0.156 | 1.000 | Normal L-shaped |
| A6 | 0.375 | 1.000 | 0.375 | Shifted |
| I1 | 0.813 | 1.000 | 1.000 | Shifted |
| I2 | 0.094 | 1.000 | 0.625 | Shifted |
| I3 | 1.000 | 1.000 | 1.000 | Normal L-shaped |
| I4 | 0.125 | 1.000 | 0.250 | Shifted |
| T1 | 0.820 | 0.301 | 0.652 | Normal L-shaped |
| T2 | 0.844 | 0.844 | 1.000 | Normal L-shaped |
| T3 | 0.078 | 0.469 | 0.297 | Normal L-shaped |
| T4 | 0.004** | 0.039* | 0.008** | Shifted |
| T5 | 0.020* | 0.074 | 0.055 | Shifted |

IAM-Infinite allele model; SMM-stepwise mutation model; TPM-(70% SMM plus 30% IAM)

*Indicates the level of significance in different mutational models
