# Supplementary material for: The level of genetic diversity and differentiation of tropical lotus, Nelumbo nucifera Gaertn. (Nelumbonaceae) from Australia, India, and Thailand
Source: Bot Stud. 2020 May 16;61:15. doi: 10.1186/s40529-020-00293-3 (PMC7229132; doi:10.1186/s40529-020-00293-3)
Supplement: Supplementary file 4 — Additional file 4: Table S4. Migrate-n results of historical gene flow among the three genetic groups. [file 40529_2020_293_MOESM4_ESM.docx]

Table S4. Migrate-n results of historical gene flow among the three genetic groups

| Parameters | 2.5% | 25.0% | Mode | 75.0% | 97.5% | Median | Mean | Nm |
| --- | --- | --- | --- | --- | --- | --- | --- | --- |
| Θ1 | 0.08220 | 0.09220 | 0.09483 | 0.09713 | 0.10000 | 0.09390 | 0.09137 | - |
| Θ2 | 0.09407 | 0.09727 | 0.09837 | 0.09940 | 0.10000 | 0.09790 | 0.09711 | - |
| Θ3 | 0.09540 | 0.09753 | 0.09850 | 0.09940 | 0.10000 | 0.09817 | 0.09759 | - |
| M_1-2_ | 0.000 | 10.000 | 20.333 | 27.333 | 43.333 | 21.000 | 20.368 | 0.494 |
| M_1-3_ | 0.000 | 8.667 | 16.333 | 23.333 | 32.000 | 17.000 | 16.019 | 0.391 |
| M_2-1_ | 0.000 | 0.000 | 4.333 | 9.333 | 20.667 | 9.667 | 4.163 | 0.095 |
| M_2-3_ | 5.333 | 16.000 | 24.333 | 32.000 | 43.333 | 25.000 | 23.631 | 0.577 |
| M_3-1_ | 0.000 | 0.000 | 5.000 | 10.000 | 21.333 | 10.333 | 4.970 | 0.121 |
| M_3-2_ | 0.000 | 8.000 | 16.333 | 23.333 | 34.667 | 17.667 | 16.312 | 0.398 |
| Mean | | | | | | | | 0.346 |

Θ-mutation-scaled population size

M-mutation-scaled migration rate

Nm-gene flow

1 Australian genetic pool

2 Indian genetic pool

3 Thailand genetic pool
